# Efficacy and safety of twice- or thrice-weekly dutasteride versus daily finasteride in men with androgenetic alopecia: A randomized, investigator-blinded, active-controlled, parallel-group pilot study

**DOI:** 10.1016/j.jdin.2025.08.009

**Published:** 2025-09-15

**Authors:** Varalee Sereepanpanich, Saranya Khunkhet, Salinee Rojhirunsakool, Montree Udompataikul

**Affiliations:** Department of Dermatology, Faculty of Medicine, Srinakharinwirot University, Bangkok, Thailand

**Keywords:** 5α-reductase inhibitors, dutasteride, intermittent regimens, low-dose dutasteride, male androgenetic alopecia, male pattern hair loss

## Abstract

**Background:**

Dutasteride is sometimes used in treating male androgenetic alopecia; however, published data on intermittent regimens are scant.

**Objective:**

To evaluate efficacy and safety of twice-weekly and thrice-weekly regimens of dutasteride, and to compare with finasteride, in men with androgenetic alopecia.

**Methods:**

Sixty men, aged 21 to 60 years, were randomized to receive dutasteride 0.5 mg twice weekly, dutasteride 0.5 mg thrice weekly, or finasteride 1 mg daily for 24 weeks. Efficacy was evaluated by changes of hair density and diameter using videodermoscopy, and global photographic assessment.

**Results:**

Hair density and diameter significantly increased after treatment in 3 groups (all *P* < .05), showing a dose-dependent manner among dutasteride groups. Mean changes of terminal hair count from baseline in twice-weekly dutasteride, thrice-weekly dutasteride, and once-daily finasteride were 7.74, 17.43, and 12.81 hairs/cm^2^, respectively. Thrice-weekly dutasteride showed a greater moderate-to-marked improvement than once-daily finasteride (35% vs 21%). Sexual adverse events were similar among 3 groups.

**Limitations:**

Unblinded participants to treatment allocation.

**Conclusion:**

Twice-weekly and thrice-weekly regimens of dutasteride increased hair growth and were well-tolerated. Although thrice-weekly dutasteride was significantly superior to twice-weekly dutasteride, in terms of hair density and diameter, thrice-weekly dutasteride did not significantly increase hair count over finasteride 1 mg daily.

**Capsule Summary**

• This randomized controlled pilot study expands the evidence of intermittent regimens of dutasteride for treating male androgenetic alopecia.
• Dutasteride 0.5 mg thrice weekly may provide an alternative therapy in men with androgenetic alopecia.

## Introduction

The 5α-reductase inhibitor (5ARI) remains central to treatments for male androgenetic alopecia (AGA) due to its effect on retro-conversion of dihydrotestosterone (DHT), the major causative hormone implicated in disease development. To treat AGA in adult men, finasteride, a selective inhibitor of 5α-reductase type II, has been approved by the US Food and Drug Administration since 1997 at 1 mg daily, whereas dutasteride, a dual inhibitor of 5α-reductase type I and II, has thereafter been approved in Japan, South Korea, and Taiwan, at 0.5 mg daily.

Finasteride and dutasteride received their first US Food and Drug Administration approvals for benign prostatic hyperplasia at a daily dose of 5 mg and 0.5 mg, respectively. The optimum daily dose of finasteride for hair loss was afterward determined at 1 mg by yielding comparable efficacy and safety to the higher 5 mg dose.^1^^,^^2^ Otherwise, dutasteride has been used at identical doses for both conditions.

From dose-ranging studies of dutasteride in male AGA, its beneficial effects on hair growth increased dose-dependently beginning at a daily dose of 0.05 to 2.5 mg, whereas a lower rate of sexual problems similarly occurred from the daily dose of 0.5 mg and under.^3^^,^^4^ Therefore, dutasteride has been usually used at 0.5 mg daily for hair loss. Moreover, dutasteride 0.5 mg daily appears superior to either finasteride 1 mg or 5 mg daily, in halting progression of hair thinning and stimulating hair regrowth, because of its greater ability to suppress DHT levels.^3^, ^4^, ^5^, ^6^, ^7^, ^8^

Despite low incidences, sexual adverse effects, including decreased libido, erectile dysfunction and reduced ejaculate volume, have always been a major concern in the use of 5ARIs among men. The strong DHT suppression of dutasteride raises the possibility of more sexual adverse events. However, evidence from well-designed clinical studies and meta-analyses demonstrates a comparable rate of sexual dysfunctions between dutasteride 0.5 mg daily and finasteride 1 mg daily.^3^, ^4^, ^5^, ^6^, ^7^, ^8^, ^9^

The use of dutasteride below the usual dose of 0.5 mg daily has been applied for male AGA in clinical practice aiming to minimize sexual side effects. Not only is the elimination half-life of dutasteride very long, approximately 5 weeks, but also it is commercially available in one strength as unsplittable softgel capsules in many countries. Intermittent administration has therefore been introduced into 2 categories: medium-dose (0.5 mg 4-5 times a week) and low-dose (0.5 mg 2-3 times a week) regimens.

However, published data on intermittent dosing of dutasteride for hair loss is very limited, only one retrospective study, and it revealed dose-dependent responses of hair regrowth in a way similar to continuous dosing.^10^ Interestingly, no sexual dysfunctions were reported in low doses of dutasteride. The purpose of this study was to assess the treatment effects of low-dose dutasteride in 2 intermittent regimens (0.5 mg twice weekly and 0.5 mg thrice weekly) for male AGA, and to compare with the standard treatment, finasteride 1 mg daily.

## Methods

### Study design

This prospective, randomized, investigator-blind, active-controlled pilot study with a 3-arm parallel group (TCTR20221106001) was conducted at Srinakharinwirot Skin Center in Thailand, from February to September 2022. A screening period of 4 weeks was followed by 24 weeks of treatment.

Block randomization with randomly selected block sizes was applied to allocate participants in a 1:1:1 ratio to receive dutasteride 0.5 mg twice weekly (DU2), dutasteride 0.5 mg thrice weekly (DU3), or finasteride 1 mg once daily (FIN7), generated using a web-based randomization service. An independent assistant performed central randomization, prepackaged study drugs and dispensed to each participant. The assigned medications were advised to be taken after meals at consistent times. Sited investigators and outcome assessors were concealed to treatment allocation until study completion.

### Study participants

Eligible Thai men aged 20 to 60 years with mild-to-moderate AGA, type IIIv to V of the Hamilton-Norwood scale, were recruited. Exclusion criteria included significant health problems, history of depression and family history of prostate or breast cancer in first-degree relatives. Additional exclusion criteria were previous use of dutasteride, use of oral finasteride within 12 months, use of other oral medications for hair loss within 6 months, use of topical medications or injections for hair loss within 3 months and hair transplantation.

### Assessments

The primary efficacy measure was change from baseline in hair count based on paired trichoscopic images. Secondary efficacy measures included change from baseline in hair diameter, overall clinical change via physician-rated global photographic assessment and participant-rated treatment satisfaction using 10-point visual analog scales.

Trichoscopic images were obtained from the vertex area in all participants at baseline and after 24 weeks of treatment, using a 50×-magnification videodermoscope (Folliscope; Anagen Corp), corresponding to image fields of 31.07 mm^2^. Hair counts and average diameters were analyzed using Folliscope 5.0 software. Landmarks on the scalp were required in repeated measurements. If natural marks were not available, a small tattoo was placed as a reference point. Hairs were categorized upon the diameter into terminal hairs (≥60 μm) and nonterminal hairs (<60 μm; intermediate and vellus hairs).

Standardized global photographs of frontal and vertex views were taken at baseline and subsequent visits, using a high-resolution digital single-lens reflex camera mounted with stereotactic head-positioning device. Two dermatologists independently graded changes in hair growth/loss on paired global photographs using 7-point rating scales (marked, moderate or mild deterioration; no change; mild, moderate, or marked improvement). The degree of concordance between 2 assessors was evaluated, and after deliberation final ratings were stated.

Adverse events were monitored on subsequent visits occurring in 8-week intervals. After taking interviews, a validated questionnaire was adopted to assess sexual problems as being sensitive issues, using the Problem Assessment Scale of the Sexual Function Inventory. Two-item and 9-item Patient Health Questionnaires were used to detect depression.

### Statistical analyses

Comparing among 3 study groups, such as baseline characteristics and adverse events, χ^2^ test was used to examine categorical data, whereas 1-way analysis of variance or Kruskal-Wallis test was used to analyze continuous data. Paired *t* test or Wilcoxon signed-rank test was used to cf paired continuous variables, including pretreatment and posttreatment hair counts and diameters. Pairwise comparisons of continuous data on efficacy between study groups were conducted using linear regression analysis. *P* < .05 were considered statistically significant.

## Results

**Fig 1:**
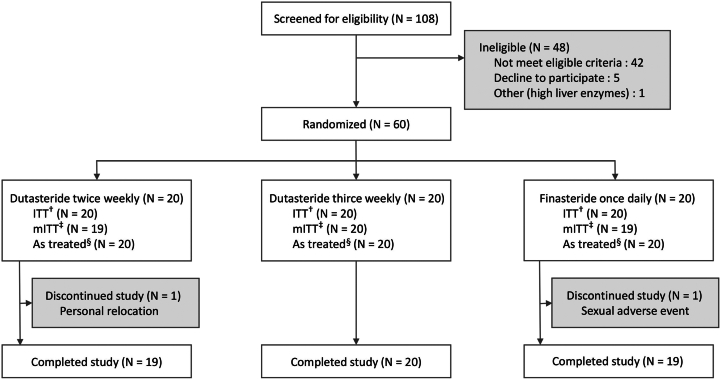
Participant disposition. *ITT*, Intention to treat; *mITT*, modified intention to treat. ^†^All randomized participants. ^‡^All randomized participants who completed study. ^§^All randomized participants who were classified according to the treatment actually received.

Participant disposition is shown in Fig 1. A total of 60 participants were randomized, and 58 participants completed the study as 2 participants dropped out at the first follow-up: one had sexual problems and the other moved abroad. Demographics and clinical characteristics of hair loss are presented in Table I. Differences in baseline characteristics, including baseline hair density and diameter, across study groups were not statistically significant in both intention-to-treat and completer populations (all *P* > .05). Medication adherence, measured by pill counts, in all groups were 97% to 99%.

**Table I:** Demographics and baseline characteristics (ITT population)

Characteristic	Dutasteride twice weekly (*n* = 20)	Dutasteride thrice weekly (*n* = 20)	Finasteride once daily (*n* = 20)
Age, y			
Mean (SD)	33.60 (8.58)	34.25 (9.04)	37.25 (11.63)
Minimum-maximum	21-53	21-53	22-60
Duration of hair loss, y			
Mean (SD)	7.15 (5.2)	7.75 (6.2)	7.05 (6.4)
Median (IQR)	5.5 (3, 10)	5.5 (3, 10)	5.0 (2.5, 10)
Minimum-maximum	0-20	1-20	1-20
Baseline Hamilton-Norwood stage, *n* (%)			
III vertex	6 (30)	7 (35)	5 (25)
IV	6 (30)	9 (45)	4 (20)
V	8 (40)	4 (20)	11 (55)
COVID-19 during the study			
No. (%)	7 (35)	9 (45)	8 (40)
Baseline hair density, hairs/cm^3^, mean (SD)			
Total hair count	99.60 (26.02)	103.14 (22.62)	106.70 (21.45)
Terminal hair count	27.00 (17.23)	36.90 (23.05)	28.85 (18.18)
Nonterminal hair count	72.60 (36.52)	66.25 (28.38)	77.85 (35.12)
Baseline hair diameter, μm			
Mean (SD)	50.74 (9.07)	52.22 (8.03)	50.65 (7.28)

*ITT*, Intention to treat.

### Changes in hair density and diameter

**Fig 2:**
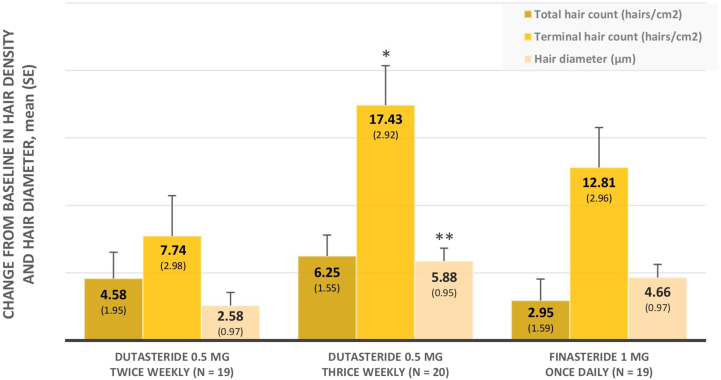
Changes from baseline in hair density and hair diameter after 24 weeks of treatment with adjustment for baseline (modified intention-to-treat population). ∗*P* = .025 and ∗∗*P* = .019 versus twice-weekly dutasteride.

After 24 weeks of treatment, mean hair counts and diameters significantly increased in all treatment groups, compared with baseline (all *P* < .05 for total hair counts; all *P* < .005 for terminal hair counts; all *P* < .01 for hair diameters). Hair density and diameter after treatment are demonstrated in Supplementary Table I (available via Mendeley at https://doi.org/10.17632/j4rhsfvfdz.3), and changes from baseline in hair density and diameter are displayed in Fig 2.

Despite no statistically significant differences, imbalance means of baseline hair characteristics were noticed among study groups. Baseline adjustment was then used in statistical models for intergroup comparisons. The highest increase in hair density and diameter after treatment was observed in DU3, followed by FIN7 and DU2, respectively. Mean changes from baseline in terminal hair counts were 17.43, 12.81, and 7.74 hairs/cm^2^, whereas mean changes from baseline in hair diameters were 5.88, 4.66, and 2.58 μm in DU3, FIN7, and DU2, respectively.

Regarding changes in terminal hair counts and hair diameters, DU3 was significantly superior to DU2 (*P* = .025 and .019). When compared with FIN7, DU3 revealed a greater increase in hair growth whereas DU2 revealed a lesser increase; however, these differences did not reach statistical significance (*P* = .23 and .27). The results also did not differ when age and disease severity were integral to the statistical model as additional covariates.

### Panel global photographic assessment

**Fig 3:**
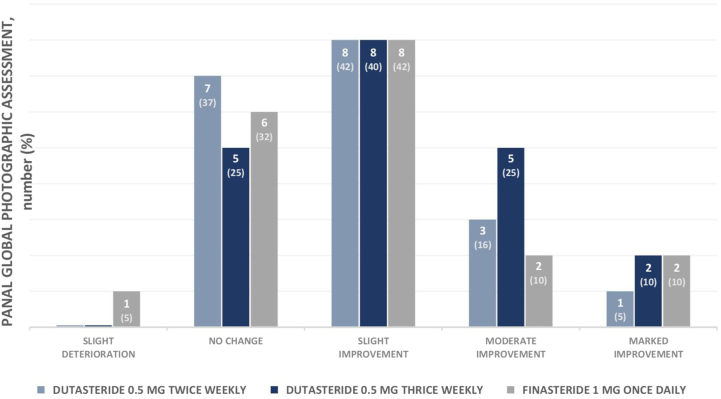
Panel assessment of overall clinical changes based on paired global photographs of the vertex and frontal views between before and after 24 weeks of treatment (modified intention-to-treat population).

There was substantial agreement on individual ratings for overall clinical changes after treatment by 2 dermatologists (kappa coefficient of 0.63). The final ratings of paired global photographs before and after treatment are exhibited in Fig 3.

After 24 weeks of treatment, over one-third of participants in all groups expressed mild improvement, representing 40% to 42%. The following portion in DU2 and FIN7 had no change (37% and 32%), whereas moderate improvement and no change were both the following portion in DU3 (25% each). Slight deterioration was observed in 1 participant (5%) in FIN7.

Clinically significant outcomes, classified as moderate-to-marked improvement, were observed in 7 participants (35%) in DU3, and 4 participants (21%) in FIN7 and DU2. Photographic representation of significant hair regrowth is shown in Supplementary Figures 1 to 6 (available via Mendeley at https://doi.org/10.17632/j4rhsfvfdz.3).

### Patient satisfaction

The scores of patient satisfaction were 7.58 ± 1.36, 7.29 ± 1.68, and 6.92 ± 1.99 in DU3, FIN7, and DU2, respectively (*P* = .487).

### Safety assessments

Adverse events were similar across 3 treatment groups, as shown in Table II. There were no statistically significant differences in incidences of overall adverse events, sexual problems and adverse events leading to study withdrawal.

**Table II:** AEs (as treated population)

AEs no. (%)	Dutasteride twice weekly (*n* = 20)	Dutasteride thrice weekly (*n* = 20)	Finasteride once daily (*n* = 20)
Discontinued treatment due to AEs			
Sexual problems	0	0	1 (5)
Participants with ≥1 AE			
Sexually related AEs	2 (10)	1 (5)	3 (15)
Decreased libido	2 (10)	0	3 (15)
Erectile dysfunction	1 (5)	1 (5)	2 (10)
Decreased ejaculation volume	1 (5)	1 (5)	2 (10)
Dizziness	1 (5)	2 (10)	1 (5)
Breast enlargement	0	1 (5)	0
Testicular pain	1 (5)	1 (5)	0

*AE*, Adverse event.

Sexually related adverse events were defined when Problem Assessment Scale of the Sexual Function Inventory score <9. During the study, sexual adverse events were revealed in 3 participants (15%) in FIN7, 2 participants (10%) in DU2, and 1 participant (5%) in DU3. One participant in FIN7 complained of significant sexual problems at the first follow-up and requested study termination. By contrast, other events were detected from self-administered questionnaires, and mild in severity without interfering in personal life. All of them were willing to continue medication. Mood disturbance was not observed in the study.

## Discussion

The benefits of daily low-dose dutasteride for hair regrowth in men with AGA have been previously demonstrated.^3^^,^^4^ After 24 weeks of treatment, dutasteride 0.02 mg daily was not different from placebo, whereas dutasteride 0.05 mg and 0.1 mg daily significantly increased hair counts and improved in global photographic assessments, compared with placebo.

The effects of dutasteride in continuous regimens on lowering DHT levels and promoting hair growth are dose-dependent.^3^^,^^4^^,^^8^^,^^11^ Interestingly, low-dose dutasteride at 0.1 mg daily appears to provide efficacy outcomes at least similar to finasteride. Olsen et al^3^ reported average increases of 78.5 and 75.6 hairs per 0.79 square inches, together with 70% and 73% reductions in serum DHT, after 24 weeks of daily treatment with dutasteride 0.1 mg and finasteride 5 mg respectively in Caucasian men. From a following study in Asian and Hispanic men, dutasteride 0.1 mg daily increased hair count by 63 hairs per 0.79 square inches and hair diameter by 3.9 μm, whereas finasteride 1 mg daily increased 56.5 hairs and 4.0 μm.^4^

Contrarily, dutasteride-associated sexual dysfunction does not appear dose-dependent in all studies. As for dose-ranging trials of dutasteride in hair loss and benign prostatic hyperplasia, decreased libido was similarly reported in daily use of dutasteride 0.01, 0.02, 0.05, 0.1, and 0.5 mg, whereas dutasteride 2.5 mg daily caused a higher rate in the trial by Olsen et al.^3^^,^^4^^,^^8^

Intermittent regimens of dutasteride have been offered in clinical practice. A retrospective study by Vañó-Galván et al^10^ elucidated effects of dutasteride monotherapy for at least 12 months in 42 men with mild-to-moderate AGA. Twelve men were treated with low-dose dutasteride in 2 intermittent regimens: 0.5 mg twice weekly and 0.5 mg thrice weekly (approximately equivalent to daily doses of 0.15 and 0.2 mg). All of 12 men had no progressive hair thinning. Eight men (66%) experienced mild improvement, whereas the rest were stable. Notably, no sexual problems were observed during treatment with twice-weekly and thrice-weekly dutasteride. However, higher doses of dutasteride were more effective. All of 30 men, treated with 0.5 mg 5 or 7 days a week, indicated hair regrowth. Marked improvement was noticed in 27% of the medium dose and 40% of the usual dose, but sexual dysfunctions occurred in both higher-dose regimens, ranging 7% to 13%.

In our study, hair counts and diameters significantly increased after 24 weeks of treatment with twice-weekly and thrice-weekly dutasteride. Thrice-weekly regimen demonstrated statistical superiority over twice-weekly regimen for improving hair growth. These findings support therapeutic effects of low-dose dutasteride used as intermittent regimens in male AGA, showing a dose-dependent fashion as continuous regimens. By comparison to daily use of finasteride 1 mg, thrice-weekly dutasteride was more effective at increasing hair density and thickness, whereas twice-weekly dutasteride was less effective than daily finasteride in this regard. These results are inconsistent with dose-ranging studies of dutasteride in continuous regimens, in which the daily use of dutasteride 0.1 mg increased more hair density than the daily use of finasteride 1 mg or even 5 mg.^3^^,^^4^ This might imply that the same total doses of continuous and intermittent regimens are not equivalent.

Regarding clinical improvement based on global photographs, consistent with previously reported retrospective data,^10^ no men treated with twice-weekly or thrice-weekly dutasteride experienced progressive hair loss, demonstrating sustained disease stabilization by low-dose dutasteride. Benefits in hair regrowth were elicited in 63% of twice-weekly dutasteride and 75% of thrice-weekly dutasteride, in parallel rates with previous data. However, we found significant hair restoration after 24 weeks of treatment, classified as moderate-to-marked improvement, in 21% of twice-weekly dutasteride and 35% of thrice-weekly dutasteride. These are conflicting with previous retrospective data, in which there was only mild improvement from low-dose dutasteride. We also found that thrice-weekly dutasteride was superior to once-daily finasteride at promoting significant hair restoration (35% vs 21%).

Although twice-weekly and thrice-weekly dutasteride were well-tolerated in this study, mild symptoms of sexual dysfunctions were found in 5% to 10%. In accordance with previous studies of dutasteride in continuous regimens, sexual adverse events from low-dose dutasteride were not different from the usual dose and finasteride.^3^^,^^4^ Moreover, sexual problems reported in this study were relatively high compared with previous large-scale studies.^1^, ^2^, ^3^, ^4^ This might be from different methods to collect data. Nocebo effects from risk warnings at informed consent are suspected to be another explanation.

Besides, the prostate-specific antigen test used for screening prostate cancers should be interpreted with caution in 5ARI users because of its lowering effects on prostate-specific antigen values by about 50%. Although long-term use of 5ARIs can reduce overall incidence of prostate cancers, US Food and Drug Administration warning is still in place for possible association with an increased incidence of high-grade prostate cancers.^12^^,^^13^ Even if serum concentrations of dutasteride can be slightly altered when taken with meals, 5ARIs can generally be administered with or without food.^14^^,^^15^ Overall, 5ARIs have no significant drug interactions of clinical importance; however, as they are primarily metabolized by the cytochrome P-450 (CYP) 3A4, caution is advised when coadministered with potent CYP3A4 inhibitors.^14^^,^^15^ Moreover, when systemic side effects of 5ARI are concerned, topical formulations can be beneficial treatment options.^16^^,^^17^

Limitations of this study included small sample size, monoethnicity, unblinded participants, and short treatment duration. Longer periods of treatment may be required to perceive the full extent of clinical responses of low-dose therapies. The small sample size can limit the ability to detect potentially significant differences in sexual dysfunction. Also, future studies should directly compare intermittent and continuous dosing of dutasteride.

In conclusion, dutasteride use in twice-weekly and thrice-weekly regimens for 24 weeks improved hair growth in male AGA with minimal side effects. Thrice-weekly dutasteride showed superior efficacy over twice-weekly dutasteride with similar adverse events. Thrice-weekly dutasteride also increased hair density and thickness to a greater extent than finasteride 1 mg daily. Dutasteride 0.5 mg thrice weekly can be a treatment option in men with mild-to-moderate AGA, especially men who aim to halt disease progression and concern about drug-related sexual problems.

## Conflicts of interest

None disclosed.
